# Deciphering the rule of antigen-antibody amino acid interaction

**DOI:** 10.3389/fimmu.2023.1269916

**Published:** 2023-12-04

**Authors:** Min Jiang, Changyin Fang, Yongping Ma

**Affiliations:** Department of Biochemistry and Molecular Biology, Molecular Medicine and Cancer Research Center, Basical Medical Collage, Chongqing Medical University, Chongqing, China

**Keywords:** antigen, antibody, interaction, antigenic drift, reverse antibody technique

## Abstract

**Purpose:**

Antigenic drift is the biggest challenge for mutagenic RNA virus vaccine development. The primary purpose is to determine the IEMM (immune escape mutation map) of 20 amino acids’ replacement to reveal the rule of the viral immune escape.

**Methods:**

To determine the relationship between epitope mutation and immune escape, we use universal protein tags as a linear epitope model. To describe and draw amino acid linkage diagrams, mutations of protein tags are classified into four categories: IEM (immune escape mutation), ADERM (antibody-dependent enhancement risk mutation), EQM (equivalent mutation), and IVM (invalid mutation). To overcome the data limitation, a general antigen-antibody (Ag-Ab) interaction map was constructed by analyzing the published three-dimensional (3D) Ag-Ab interaction patterns.

**Results:**

(i) One residue interacts with multiple amino acids in antigen-antibody interaction. (ii) Most amino acid replacements are IVM and EQM. (iii) Once aromatic amino acids replace non-aromatic amino acids, the mutation is often IEM. (iv) Substituting residues with the same physical and chemical properties easily leads to IVM. Therefore, this study has important theoretical significance for future research on antigenic drift, antibody rescue, and vaccine renewal design.

**Conclusion:**

The antigenic epitope mutations were typed into IEM, ADERM, EQM, and IVM types to describe and quantify the results of antigenic mutations. The antigen-antibody interaction rule was summarized as a one-to-many interaction rule. To sum up, the epitope mutation rules were defined as IVM and EQM predomination rules and the aryl mutation escape rule.

## Introduction

Because RNA polymerase lacks the error-correcting mechanism of 5’-3’ exonuclease and causes the genetic variation of the virus (1), when this mutation produces amino acid substitution in the neutralizing antigen (Ag), it leads to typical antigenic drift and immune escape (2). An RNA virus usually undergoes antigenic drift. The antigenic drift successful model states that mutation can continuously produce new strains (3). However, the majority of these are unable to proliferate within the host population because of pre-existing immune responses directed against epitopes with restricted diversity. Once the immunodominant epitope of the virus surface protein is mutated to form a new subtype, the existing neutralizing antibody (Ab) no longer neutralizes the mutated virus (4). For example, an error-prone replication mechanism in influenza viruses results in antigenic drift and viral escape from the immune response which also leads to seasonal and pandemic diseases (5). Antigenic drift poses a serious problem in vaccine development and updating. During the severe acute respiratory syndrome coronavirus 2 (SARS-CoV-2) epidemic, antigenic drift occurred frequently (6–9).

For instance, SARS-CoV-2 has high genetic variability and rapid evolution (10, 11). Particularly, natural selection has a tendency for specific mutations., *e.g.*, E484K has a mutation frequency of 5.5, which is five times greater than E484Q; it shows that E484K is more frequently detected in the population (12). Because the SARS-CoV-2 mutants in the current epidemic are resistant to neutralizing Abs, how to solve antigenic drift is a substantial theoretical and practical problem (6). The strains B.1.617.2 and B.1.1.529 have swept the world and led the virus to evade the immune response (13–17). This has forced the redesign and production of new vaccines to cope with the new variants (18). However, the dilemma is that vaccine development cannot keep pace with viral mutations. Consequently, identifying how to understand amino acids in the context of Ag-Ab interaction and growing a wide-spectrum vaccination or rescuing monoclonal antibody (mAb) is of extreme importance.

Immune recognition occurs in matching and anastomosis between specific positions and specific fragments of Ab and Ag molecules. Due to the complex spatial structure of proteins and the diversity of organisms, it is very difficult to predict exactly how the antigen-determined amino acid will mutate. Thus, exploring the rule of amino acid interaction between Ag and Ab, and then summarizing the interaction (recognition and binding) rule of amino acids for current virus immunity and vaccine preparation is of great significance. The best way is to detect changes in the ability of the antigen to bind to the mAb by mutating the key amino acid on the epitope to summarize the regular amino acid interaction spectrum.

We used linear epitopes to study antigens because antigen spatial epitopes are complex. To describe the relationship between linear epitope mutation and immune escape, we cautiously assumed four concepts: (i) Immune escape mutation (IEM) meant that the residue substitution caused the antigen to lose its affinity (recognition) to pre-existing Ab or to remain less than the assumed 30% affinity without neutralization. (ii) Antibody-dependent enhancement risk mutation (ADERM) refers to the residue substitution causing the antigen to remain a pre-existing Ab affinity of more than the assumed 30% but less than 40%. However, the pre-existing Abs could not neutralize the mutated antigen (pathogen). Rather, the virus-Ab complex with low affinity enhanced virus uptake resulting from the attachment of immune complexes to the Fcγ receptor and enhanced the infection. (iii) Equivalent mutation (EQM) meant that the residue substitution led the Ag to remain at pre-existing Ab affinity beyond the assumed 40% but less than 80%. Fortunately, the pre-existing Ab still completely neutralizes the antigen. (iv) Invalid mutation (IVM) indicated that the residue substitution did not affect the pre-existing Ab affinity and the Ab completely neutralized the Ag. The quantitative criteria were determined according to the referenced literature (19, 20). The method of enzyme-linked immunosorbent assay (ELISA) was used to measure binding affinity between anti-protein tag mAbs and protein tag mutants. To address the data limitation, we also created an Ag-mAb interaction pairs summary presented with 3D structures by literature search.

## Materials and methods

### Peptides and agents

Peptides of HA-tag, c-MYC-tag, VSV-tag, Flag-tag, and their mutants (more than 98% purity) were synthesized commercially by Nanjing Yuanpeptide Biotech (Nanjing, China) (**Tables S1**-**S4**). The selection criteria for the universal protein tags were to get the peptide sequence as short as possible, which made it easier and more cost-effective to synthesize. Secondly, the amino acid sequence of each tag was diverse as it would cover 20 amino acids. As a result, the residues in the HA-tag were not duplicated when replacing the c-MYC-tag, VSV-tag, or Flag-tag. Additionally, mAbs that recognized the tags were easy to purchase and evaluate and were sensitive to the wild type and its mutations. To increase the solubility and binding ability of the universal protein tags in the 96-well plate, random peptides of 18 amino acids were respectively fused with the target tag at the C-terminus via the GSGSGS linker. Mouse anti-HA-tag mAb labels were purchased from Qrigene cat# TA180128, MD, USA. Mouse anti-c-MYC-tag and anti-Flag-tag mAb labeled were purchased from Proteintech cat# 6003-2-lg/66008-3-lg, Wuhan, China. Mouse anti-VSV-tag mAb labeled were purchased from Abbkine Scientific cat# A02180, Wuhan, China. HRP-conjugated AffiniPure goat anti-mouse IgG from Proteintech, cat# SA00001-15, Wuhan, China, and DAB color development kit cat# AR1026 were purchased from a Biological company, and the other additional buffers were prepared in our laboratory.

### Enzyme-linked immunosorbent assay

The peptides of tags and their mutants were diluted to 2.0 μg/ml and 50 μl each was added into 96 well plates for 1 hour to adsorb (n=3). After washing with phosphate buffer saline containing 0.05% Tween-20 (PBST) three times, the plate was blocked with a rapid-blocking buffer for 10 min. After washing with PBST three times, mouse anti-tags mAbs were added into each well (40 μl/well) and incubated at 37°C for 1 h. Next, after washing and blocking as described above, 100 μl of horseradish peroxidase (HRP)-labeled goat anti-mice IgG (1:2500) were added and incubated at 37°C for 1 h. The results were detected by the plate reader after color-developing with TMB single-component substrate solution (Solarbio, cat# PR1200, Beijing, China) and stopping. Peptide-free and mAb-free wells were performed as negative controls. The absorbance value at 450 nm (OD_450_) was measured for analysis.

### Literature 3D-structure references and amino acid-amino acid interaction assay

A literature search was performed using the keywords: ‘protein ‘interaction’, ‘antigen-antibody ‘interaction’, and ‘crystal structure of binding ‘surface’ in the NCBI PubMed database (https://www.ncbi.nlm.nih.gov/pubmed/) and Web of Science (https://www.webofscience.com/wos/alldb/basic-search) to collect the amino acid recognition binding data documented in the literature. First, each letter was carefully read and the computer-predicted Ag-mAb model was extracted. Only the models established by X-ray or cryo-electron microscopy with clear textual identifications were included in the statistical analysis along with specific interaction patterns between amino acids.

All collected data were entered into Microsoft Excel™ and visualized with Cytoscape and GraphPad Prism 8.02 software. After partial mutation, the mutant polypeptide sequence and the original protein-tag sequence of IVM and IEM were generated using the online prediction software PEP-FOLD3 (https://bioserv.rpbs.univ-paris-diderot.fr/services/PEP-FOLD3/) and AlphaFold (https://github.com/google-deepmind/alphafold/tree/main/alphafold), and a 3D structure was obtained. The best coupling model was selected according to the score. At the same time, the PDB file was downloaded to UCSF Chimera X, and the Matchmaker program was performed on the 3D structure of the mutant and the original protein tag to obtain the structure distance RMSD (root mean square deviation) value and the 3D conformational map.

## Results

### Results of protein-tags ELISA

**Figure 1:**
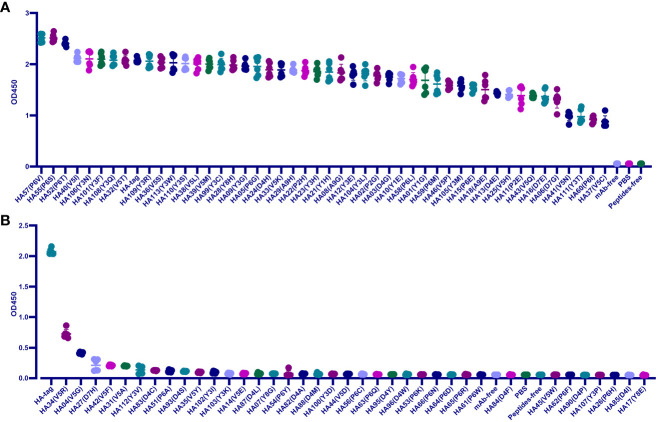
Scanning the binding affinity of 84 HA-tag mutants to mAb TA180128. **(A)** Forty-seven HA-tag mutants maintained more than 40% mAb binding affinity. These were EQM and IVM mutants. **(B)** HA (V5R) mutant was ADERM for 35% mAb binding affinity. The other 36 HA-tag mutants with a mAb binding affinity of less than 20% were IEM. P<0.0.5.

We screened the key residues in HA-tag to mAb TA180128. At first, the HA-tag (Y1P2Y3D4V5P6D7Y8A9) was substituted one by one from Y1 to A9 with G, E, or H, respectively. Except for the A9 residue, mAb TA180128 reacted to the other residues from Y1 to Y8 in this study. After substituting with other 19 amino acids from Y1 to Y8, we built a total of 84 HA-tag mutants. The 32 (38.1%) mutants were categorized as IVM (P6V, P6S, P6T, V5I, Y3N, Y3F, Y3Q, V5T, Y3R, V5S, Y3W, Y3S, V5L, V5M, Y3C, Y8H, Y3G, P6G, D4H, V5K, A9H, P2H, Y3H, Y1H, A9G, Y3E, Y3L, P2G, D4G, Y1E, P6L, and Y1G) for maintaining more than 80% affinity to mAb TA180128. The 15 mutants (17.8%) were EQM (P6M, V5P, Y3M, P6E, A9E, D4E, V5H, P2E, V5Q, D7E, D7G, V5N, Y3T, P6I, and V5C) for maintaining 43%- 77% affinity (**Figure 1A**). The HA (V5R) mutant was ADERM for maintaining 35% mAb-binding affinity. The other 36 mutants (42.9%) were IEM (V5G, D7H, V5F, V5A, Y3V, D4C, P6A, D4S, V5Y, Y3I, Y3K, V5E, D4L, Y8G, P6Y, D4A, D4M, Y3D, V5D, P6C, P6Q, D4W, D4Y, P6K, P6N, P6D, P6R, P6W, D4F, V5W, P6F, D4P, Y3P, P6H, D4I, and Y8E) for losing the affinity to mAb TA180128 completely (**Figure 1B**). However, P6V, P6S, and P6T mutants were found to have 16%-21% superior affinity compared to their parental HA-tag (**Figure 1A**). Therefore, in 84 HA-tag mutants, approximately 55.9% of antigenic drift (IVM, 38.1% and EQM, 17.8%) did not affect the immunity of mAb TA180128 (**Figure 1A**).

Since IVM and EQM did not affect the protective effect of the current vaccine, the aim was to reveal the IEM of Y3, D4, V5, P6, D7, and Y8 substitutions replaced by the other 19 amino acids, respectively. In summary, V5, P6, D4, and Y3 were IEM hotspots, accounting for 24.1%, 37.9%, 31%, and 17.2% of IEM, respectively (**Figure 1B**). Most of the D7 and Y8 residues were IVM and EQM hotspots, and only D7H and Y8E mutations were IEM (**Figure 1**).

To cover 20 amino acid substitutions, we added the MYC-tag, Flag-tag, and VSV-tag to the experimental catalog. Those amino acids that did not repeat on the HA-tag were selected for mutation. Specifically, we replaced the Q2, S6, and E8 residues of MYC-tag with G and its L4 and I5 with other 19 amino acids, respectively. While the other 19 amino acids replaced the K3 of Flag-tag, G replaced the N7, R8, and I4 in VSV-tag, and the T2 and M6 were mutated to the other 19 amino acids, respectively.

**Figure 2:**
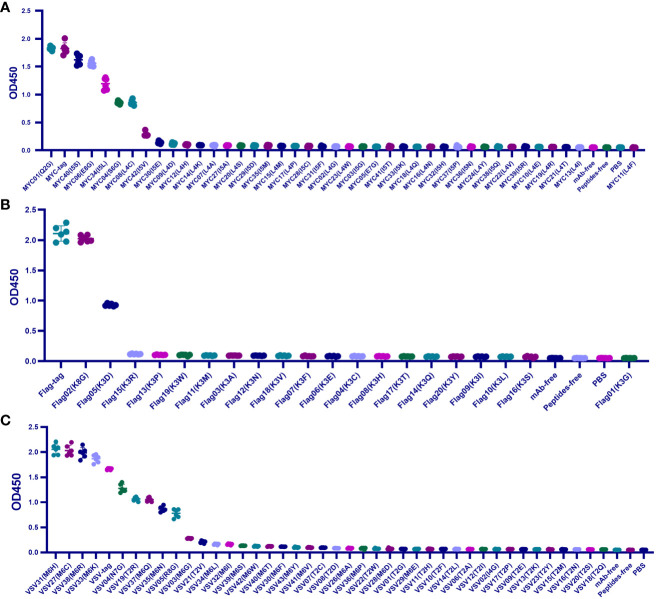
Scanning the binding affinity of MYC-tag, Flag-tag, and VSV-tag mutants. **(A)** Scanning the binding affinity of 40 MYC-tag mutants of Q2X, L4X, I5X, S6X, and E8X to mAb6003-2-lg. IVM and EQM were represented by means of the mutants Q2G, I5S, E8G, I5L, and S6G; IEM was represented by the remaining 36 mutants. **(B)** Analyzing the mAb 66008-three-lg binding affinity of 20 Flag-tag mutants K3X. EQM became K3G, even as IVM became K8D. **(C)** Measuring the binding affinity of 41 VSV-tag mutants of T2X, M6X, N7X, R8X, and I4X to mAb A02180. The ultimate 18 replacements were IEM. M6C, M6H, and M6K were IVM mutations. EQM blanketed the mutants M6R, N7G, T2R, M6Q, M6N, and R8G. The last 30 mutants were IEM contributors. P < 0.05.

For 39 c-MYC-tag mutants, except for the affinity of Q2G, I5S, and E8G > 80%, which belonged to IVM, I5L and S6G belonged to EQM for maintaining 65% and 47% affinity. The other 34 mutants had less than 20% affinity or could not be combined with mAb and were classified as IEM (**Figure 2A**).

Among the 20 mutants of Flag-tag, only K8G shared 95.9% mAb-binding affinity with Flag-tag and was defined as IVM, and K3D maintained 44% mAb-binding affinity and was defined as EQM. The other 18 K3 mutants were IEM for having less than 6% affinity (**Figure 2B**).

Similarly, among the 41 mutants of Flag-tag, M6C, M6H, M6K, and M6R of VSV-tag were IVM for maintaining more than 110% affinity compared to VSV-tag. N7G, T2R, M6Q, M6N, and R8G were EQM for maintaining 47%-77% affinity. The other 32 mutants were IEM for having less than 17% affinity (**Figure 2C**).

Unexpectedly, in total, the 184 universal protein tags, HA(P6V), HA(P6S), HA(P6T), VSV (M6C), VSV (M6H), VSV (M6K), VSV (M6R), and MYC (Q2G) replacement were enhanced by mAb-binding affinity from 112% to 121%, respectively (**Figures 1A**, **2**).

**Figure 3:**
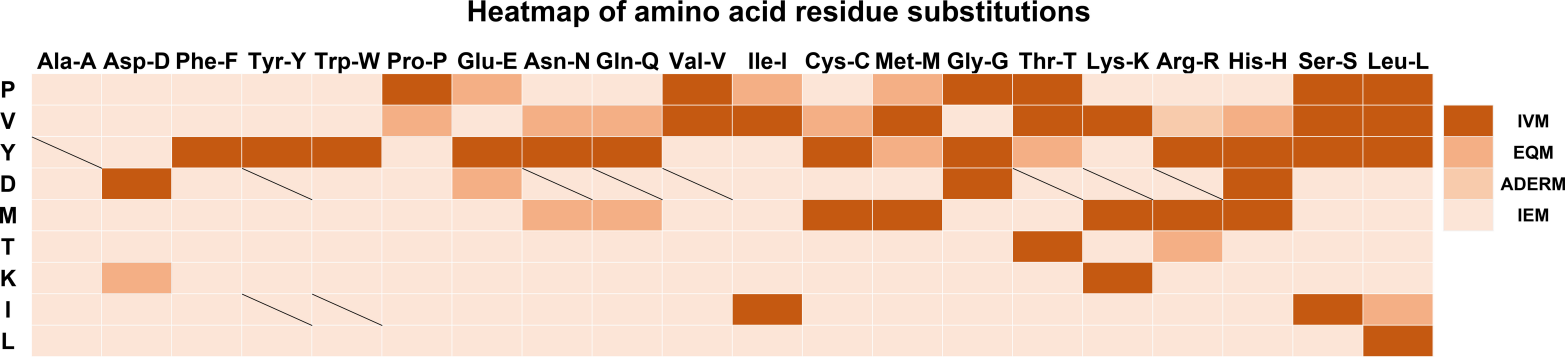
Heatmap of amino acid residue substitution verified by ELISA. The vertical lines represent the amino acids of the universal protein tags. The horizontal amino acid was used to replace the original amino acids of the universal tag, respectively. Heatmap analysis suggested that most of the replacements were IEM. The slash line indicates that no experimental data were available. IVM, Invalid mutation; EQM, Equivalent mutation; ADERM, antibody-dependent enhancement risk mutation; IEM, Immune escape mutation; P, HA-P6; V, HA-V5; Y, HA-Y3; D, HA-D4; M, VSV-M6; T, VSV-T2; K, Flag-K3; I, MYC-I5; L, MYC-L4.

**Figure 4:**
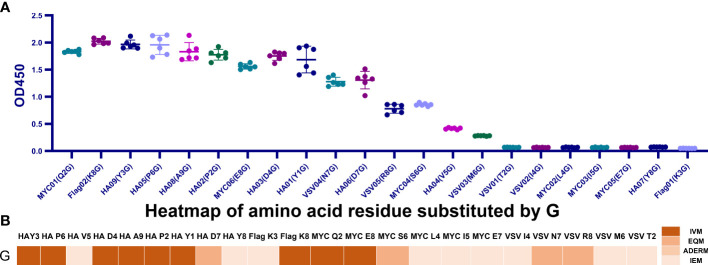
All G-replaced mutants’ binding affinity maps over four tags. **(A)** Examining each G-replaced mutant’s binding affinity map across four tags. MYC (L4G), MYC (I5G), MYC (I4G), MYC (E7G), HA (Y8G), VSV (T2G), and Flag (K3G) were IEM, and the remaining 15 mutants belonged to IVM and EQM. **(B)** Heatmap of the affinity of the G-replaced mutant tags. Approximately 59.1% of replacements were IVM and EQM.

To display the mutant interaction visually, we used Microsoft Excel to draw the heatmap of amino acid residue substitution in this study. From the selected and substituted nine amino acids, I, K, L, and T tended to lose their mAb affinity after substituting them with the other 19 amino acids (**Figure 3**) except that the binding affinity substituted by C, H, K, and R could be maintained, and substituting M by other amino acids led to IEM. Similarly, when E, H, and G replaced D, it could still recognize and bind to mAb. The other substitutions of D led to IEM. Conversely, approximately 70% of substitutions of V and Y showed IVM. Most substitutions of V and Y retained their original immune affinity. Only V/A, V/D, V/F, V/Y, V/W, V/E, V/G,Y/D, Y/P, Y/V, Y/I, and Y/K replacement models produced IEM (**Figure 3**). Nevertheless, only 30% of substitutions of P showed IVM. P/E, P/I, and P/M showed EQM, and the other 11 substitutions were IEM (**Figure 3**). Notably, all amino acids replaced by A, D, F, P, W, and Y led to IEM, and 98% of amino acids replaced by V and I might be IEM (**Figure 3**). However, 66% of amino acids replaced by G might be IEM (**Figure 4**).

### Literature data analysis of antigen-antibody interactions

**Figure 5:**
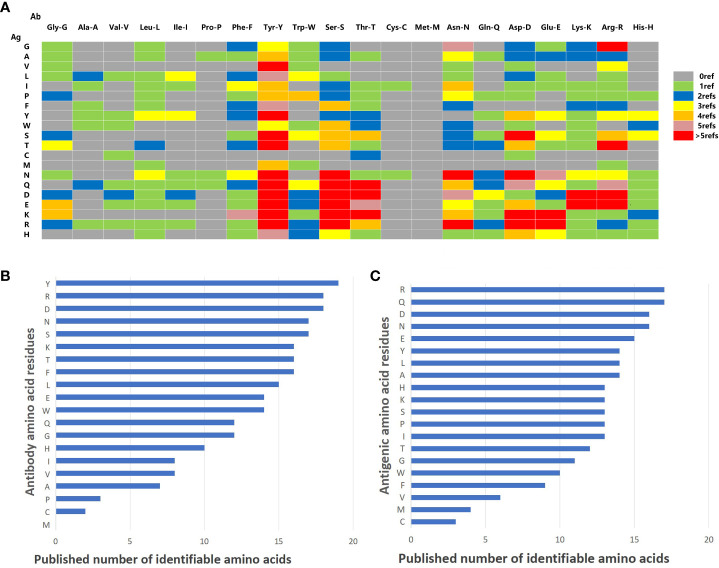
Summary of literature amino acid recognition rules. **(A)** Amino acid recognition rule matrix collected by literature. The vertical lines represent amino acids on Ag. The horizontal lines represent the amino acids on mAb. **(B)** Histogram of identifiable amount of amino acid residues in antibodies. **(C)** Histogram of identifiable quantity of amino acid residues in antigen.

Seventy articles related to Ag-Ab interaction were screened through the NCBI PubMed (https://pubmed.ncbi.nlm.nih.gov) and Web of Science (https://access.clarivate.com). The interactions of amino acid residues at the Ag-Ab binding interface were extracted from the 3D structures reported in the literature, and the amino acid binding matrix was drawn and colored according to the number of relevant literature (**Figure 5A**). The X-axis is the amino acids of the Ab, and the Y-axis is antigenic residues. According to the matrix, the binding rules of amino acids in Ags and Abs tended to be consistent. Among the 20 types of amino acids, Y, S, D, and R had an affinity with other amino acids and could recognize almost all amino acids in the literature. However, C and M were practically silent and only interacted with relatively few amino acids, coinciding with some of our ELISA experiments’ findings. Explicitly, residue M was not found in the Ab interface and only four were found in the Ag interface (**Figures 5B, C**). Residue C was found only one in the Ab interface and only three were found in the Ag interface (**Figures 5B, C**). Otherwise, P was rarely found in Ab interfaces and V was rarely found in Ag interfaces (**Figures 5B, C**).

Next, the histogram of the interaction capacity of the 20 amino acids was plotted according to the literature. The top 10 amino acids with the highest Ab detection rate were Y, R, D, N, S, K, T, F, L, and E, respectively (**Figure 5B**). However, the top 10 detection amino acids in Ags were R, Q, D, N, E, Y, L, A, H, K, S, P, and I (**Figure 5C**), which explains that H, K, S, P, and I shared the 10th rank for their same detection rate (**Figure 5C**).

### Amino acid interaction network analysis

**Figure 6:**
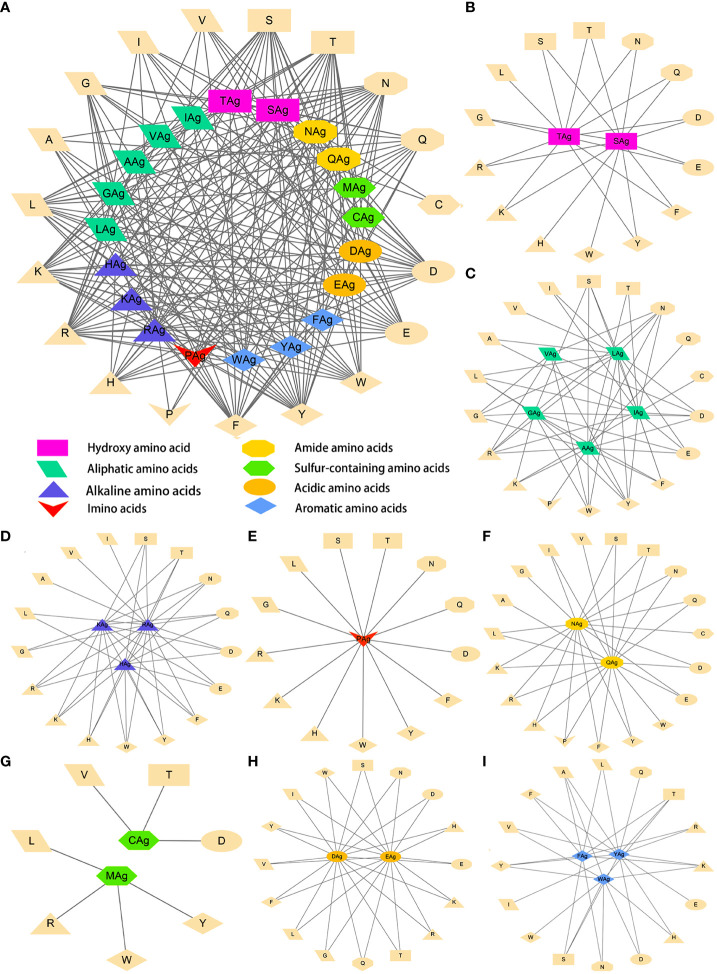
Literature antigen-antibody amino acid interaction diagram. **(A)** Summary diagram of Ag-Ab amino acid interaction based on physical and chemical properties. **(B)** Diagram of interactions between hydroxyl-amino acids and 20 amino acids. **(C)** Diagram of interactions between aliphatic amino acids and 20 amino acids. **(D)** Diagram of interactions between alkaline amino acids and 20 amino acids. **(E)** Imino acids. **(F)** Amide amino acids. **(G)** Sulfur-containing amino acids. **(H)** Acidic amino acids. **(I)** Aromatic amino acids. The antigenic amino acid abbreviation was labeled with the subscript Ag to distinguish antibody amino acids.

**Figure 7:**
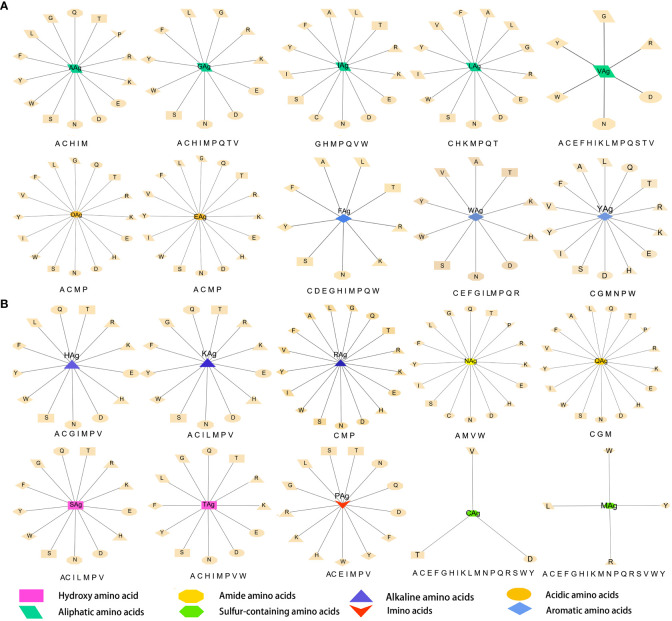
Twenty antigenic amino acid interactions were plotted individually. Antibody amino acids without interaction are listed in bold type under the main figure, respectively. The antigenic amino acid abbreviation was labeled with the subscript Ag to distinguish antibody amino acids.

The data were imported into Cytoscape to draw an interaction network. According to the physical and chemical properties, 20 amino acids were divided into eight categories, which were aliphatic amino acids, aromatic amino acids, acidic amino acids, alkaline amino acids, amide amino acids, sulfur-containing amino acids, hydroxyl amino acids, and imino acids. Then, the interaction diagram was constructed (**Figure 6A**). Except for a few aliphatic amino acids and all sulfur-containing amino acids, the side chains of hydroxyl-amino acids S and T could interact with 14 amino acids in the literature (**Figure 6B**). The aliphatic amino acids included A, V, I, and L, and only I could interact with C (**Figure 6C**). The alkaline amino acids K, R, and H could interact with 17 kinds of amino acids in Abs (**Figure 6D**). Notably, antigenic P interacted with 13 amino acids in Abs, and P in Abs only interacted with antigenic N, Q, and A (**Figures 6A, C, E, F**). The antigenic C (interacted with D, T, and V) and M (interacted with L, R, W, and Y) contained Sulphur in their side chains, causing them not to cross-link (**Figure 6G**). Otherwise, the residue of Ab C only recognized antigenic I and N (**Figures 6A, C, F**). Acidic amino acids could interact with 16 amino acids (**Figure 6H**). Similarly, antigenic aromatic amino acids seemed to interact with more than 10 amino acids (**Figure 6I**). To quickly view the amino acid interaction between Ag-Ab, single interaction networks were plotted individually in the alphabetical order of amino acid abbreviations. Amino acids without interaction were listed below the main figure in boldface (**Figure 7**).

### Verification of experimental data and literature data

**Figure 8:**
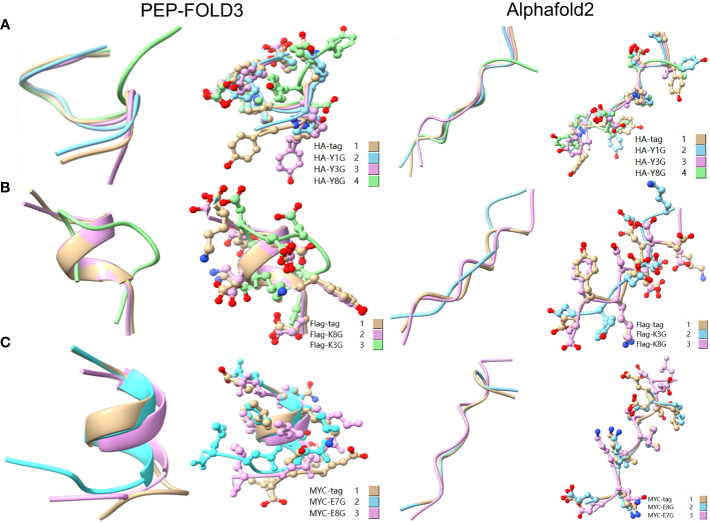
Comparison of the 3D structure of mutant tags replaced with G. **(A)** Comparison of HA-tag with HA-Y1G, HA-Y3G, and HA-Y8G **(B)** Comparison of Flag-tag with Flag-K8G and Flag-K3G **(C)** Comparison of MYC-tag with MYC-E8G and MYC-E7G. The models were predicted using the software PEP-FOLD3 and AlphaFold.

To reveal the effect of mutation on the peptide structure and verify the experimental results, we used the computer ab initio folding algorithm to predict the 3D structure of peptide tags and their mutants. We predicted the 3D structure of HA-tag, MYC-tag, and Flag-tag and their mutants (HA-Y1G, HA-Y3G, HA-Y8G, MYC-E8G, MYC-E7G, Flag-K8G, and Flag-K3G), respectively, by online tools PEP-FOLD3 and AlphaFold. After obtaining the PDB file, we compared the mutant with the original structure. The fit degree of HA-Y8G and MYC-E7G to the original tag structure was lower than other mutants (**Figure 8**). Surprisingly, the Flag-K3G mutation disrupted the original α-helix and curled into a semi-O-ring in the opposite direction of the original Flag-K3 peptide, affecting Ab binding capacity (**Figures 2B**, **S2**). Consequently, the low fit mutation resulted in IEM due to loss of interaction. The same IEM mutations happened in HA-D4F, HA-D4W, HA-D4Y, HA-V5F, HA-V5W, HA-V5Y, HA-P6F, HA-P6W, HA-P6Y, VSV-M6F, VSV-M6Y, and VSV-M6W (**Figures 10**).

**Figure 9:**
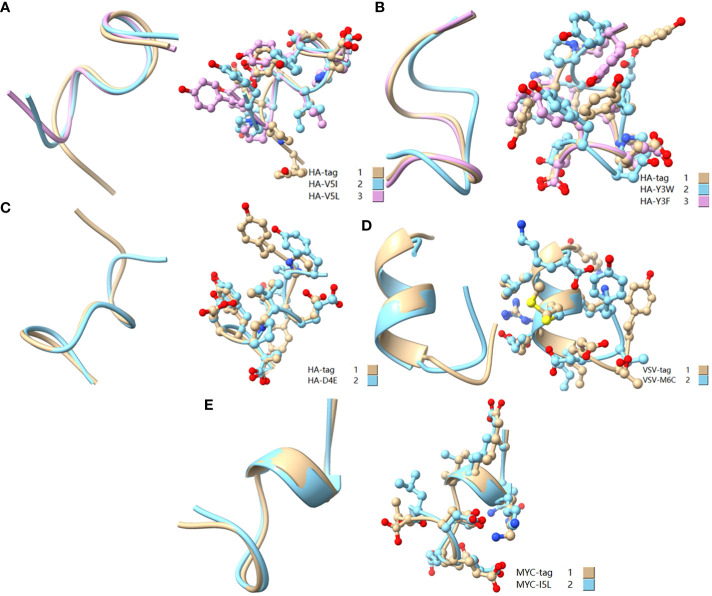
Comparison of amino acid mutant with similar physicochemical property residues. **(A)** Comparison of HA-tag, HA-V5I, and HA-V5L; **(B)** Comparison of HA-tag, HA-Y3W, and HA-Y3F; **(C)** Comparison of HA-tag and HA-D4E; **(D)** Comparison of VSV-tag and VSV-M6C; **(E)** MYC-tag and MYC-I5L. The models were predicted using the software PEP-FOLD3.

**Figure 10:**
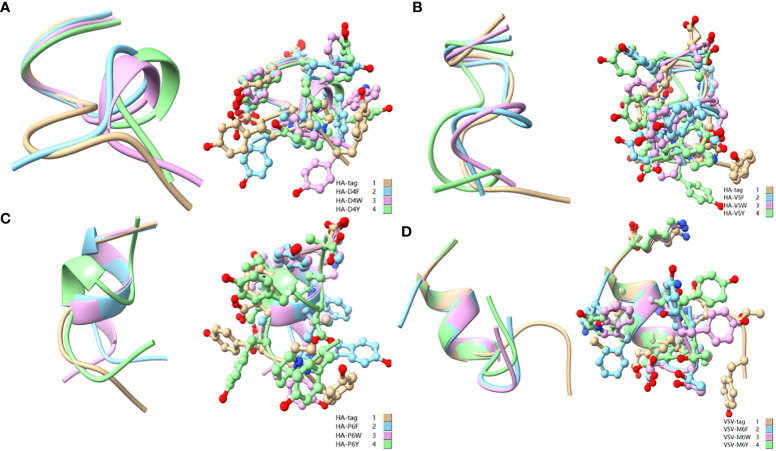
Comparison of aromatic amino acid substitution mutants. **(A)** Comparison of HA-tag, HA-D4F, HA-D4Y, and HA-D4W; **(B)** Comparison of HA-tag, HA-V5F, HA-V5Y, and HA-V5W; **(C)** Comparison of HA-tag, HA-P6F, HA-P6Y, and HA-P6W; **(D)** Comparison of VSV-tag, VSV-M6F, VSV-M6Y, and VSV-M6W. The models were predicted using the software PEP-FOLD3.

As a broadly known concept, protein tags mutation experiment found that the substitution of amino acids with similar physical and chemical properties residue tended to IVM, e.g., aliphatic amino acids V, I, and L substituted each other (HA-V5I, HA-V5L; MYC-I5L) (**Figures 1**, **2A, C**). Similarly, aromatic amino acids Y, W, and F mutual substitution were also IVM, *e.g*., HA-Y3F and HA-Y3W (**Figure 1**). The results indicated that the amino acids with the same physical and chemical properties of the side chain group shared high similarity in 3D structures (**Figure 9**). In addition, the non-aromatic amino acids were replaced by aromatic amino acids (F, Y, and W) and often lost their affinity to antibodies (**Figures 1**, **2**). It might be related to the benzene ring on the side chain of aromatic amino acids. We consequently predicted the structure of some non-aromatic amino acid mutants replaced by aromatic amino acids and compared their structural changes (**Figure 10**). To visually describe the structural changes of the mutated tags, the RMSD values were obtained in UCSF ChimeraX for the fitted structures of the above-mentioned mutants and the original protein tags were summarized (**Table 1**). If the RMSD was less than 3 Å, the two structures were considered similar. Conversely, if the RMSD was more significant than 3 Å, the two structures did not match. Thus, our test suggested that the protein-tag mutants with RMSD > 3.0 Å tended to IEM (**Table 1**).

**Table 1:** RMSD and the mAb binding affinity of the fitted structures of mutant strains and wild-type tag proteins.

Mutant	*RMSD(Å)	#RMSD(Å)	mAb binding Affinity(%)
HA-Y1G	0.776	0.810	81.43%
HA-Y3G	1.594	1.446	94.89%
HA-Y8G	4.496	3.369	3.59%
MYC-E8G	2.656	0.632	85.11%
MYC-E7G	3.835	4.367	3.59%
Flag-K3G	4.682	3.894	2.43%
Flag-K8G	0.408	1.937	95.93%
HA-V5L	1.825	0.459	95.52%
HA-V5I	1.885	0.650	96.38%
HA-Y3F	0.332	0.739	101.43%
HA-Y3W	2.457	1.601	97.94%
HA-D4E	1.845	0.623	69.24%
VSV-M6C	2.799	2.530	122.37%
MYC-I5L	0.557	0.355	65.28%
HA-D4F	4.139	3.167	2.74%
HA-D4W	3.401	3.460	3.03%
HA-D4Y	4.301	3.216	3.01%
HA-P6F	3.792	3.853	2.71%
HA-P6W	4.189	3.258	2.79%
HA-P6Y	4.214	4.053	3.58%
HA-V5F	4.760	3.927	98.79%
HA-V5W	3.509	3.803	100.57%
HA-V5Y	3.650	3.336	99.71%
VSV-M6F	4.550	3.649	6.94%
VSV-M6W	4.212	3.575	7.41%
VSV-M6Y	4.175	3.667	6.14%

*: RMD(Å) predicted in PEP-FOLD3. #: RMD(Å) predicted in AlphaFold. High consistency RMD (Å)) shared between PEP-FOLD3 and AlphaFold.

## Discussion

Viral immune escape caused by antigenic drift has always been a great challenge for vaccination and prevention (21). This study aims to establish the rule of the amino acid interaction during Ag-Ab recognition and use this rule to guide scientists to update vaccines or rescue ineffective mAb due to antigenic mutations in clinical applications. Ab rescue refers to when an essential antigenic amino acid mutation causes the failure of mAb in clinical application; the original amino acid on the mAb is replaced with a new amino acid according to the Ag-Ab recognition rule to recognize and neutralize the mutated antigen again. We termed it the reverse antibody technique. For instance, the E484K mutation of SARS-CoV-2 RBD (receptor-binding domain) disabled mAb P17 neutralization (22, 23). Three-dimensional data demonstrated that the negatively charged E484 interacted with positively charged H35, H99, and R96 residues, which formed a strong electrostatic interaction (23). Thus, the E484K mutation was repulsive to the positively charged binding site of the mAb P17. Guided by our reverse antibody theory, mAb P17 might be theoretically rescued by mutating part or all of the H35, H99, and R96 into negatively charged residues such as E and/or D.

The IEM rule obtained from protein tags was confirmed in the real-world data. For example, HA-tag P6L mutation resulted in IEM (**Figure 1**). In SARS-CoV, the P462L mutation led to escape from mAb CR3014 (24). In addition, the spike L452R mutation conferred SARS-CoV-2 escape from the immune system (25). Our findings suggested that Ags were often not recognized by Ab when other residues replaced antigenic residues K, L, or T. Correspondingly, the A180V mutation in the influenza virus promotes the virus to escape from Ab-based immunity (26–28). V483A mutation in SARS-CoV-2 was resistant to some neutralizing antibodies (29). In this study, the mutations of V to A led to IEM (**Figure 1**). The escape caused by these mutations is consistent with the escape rules we found in this study. The amino acid mutation type of Ag and the corresponding IEM pattern are termed immune escape mutation map (IEMM). IEMM establishment aims to deal with antigenic drift and immune escape of human viruses. However, IEMM is currently just a hypothesis we proposed and need to prove and refine in the future.

Theoretically, substituting epitope key residues seems to be a random event, but it has its rules. Many mutations do not exist in nature for immune pressure (30). That is, all strains found to have mutations in neutralizing antigens are immune escape strains. In particular, the single nucleotide mutations of L452 in SARS-CoV-2 are limited to Q, M, and R in nature (4, 25, 31, 32). Tan et al. characterized all 19 possible mutations at L452. They revealed that five mutants (Q, K, H, M, and R) gained greater infectivity and immune escape. The other mutants failed to maintain expression or pseudovirus infectivity (33). According to our findings, these failed mutations were either IVM or EQM. We believe that IVM and EQM are important factors and subject matter for pathogen-neutralizing antigen gene mutations. Taking the statistical analysis of SARS-Cov-2 RBD high-frequency mutation sites as an example, the proportion of IVM and EQM rate comparisons to all mutations is as high as 75% (**Table 2**).

**Table 2:** The predominance of IVM and EQM in SARS-Cov-2.

Strain	417	Result	484	Result	501	Result	Antibody affinity %
Original	K (AAG)	Immune	E (GAA)	Immune	N (AAT)	Immune	100
Alpha	K	Immune	E	Immune	Y (TAT)	IEM	42
Beta	N (AAT)	IEM	K (AAA)	IEM	Y (TAT)	IEM	22
Gamma	T (ACG)	IEM	K (AAA)	IEM	Y (TAT)	IEM	26
delta	K	Immune	E	Immune	Y (TAC)	IEM	29
Omicron	N (AAC)	IEM	A (GAG)	IEM	Y (TAT)	IEM	~9
Other theoretical mutants	K (AAA), M (ATG), R (AGG), stop (TAG), L (TTG), S (TCG), W (TGG)	(IVM+EQM)/ IEM=6/3. (IVM+EQM)/ mutations =6/9 (67%).	Stop (TAA), Q (CAA), V (GTA), A (GCA), G (GGA), D (GAT), D (GAC)	(IVM+EQM)/ IEM=6/2. (IVM+EQM)/ mutations =6/8 (75%).	stop (TAG), stop (TAA), H (CAT), D (GAT), I (ATT), T (ACT), S (AGT)	(IVM+EQM)/ IEM=5/2. (IVM+EQM)/ mutations =5/7 (71%).	

* Stop mutations were not included in IVM and EQM.

The analytical protocols in this study greatly contributed to the understanding and explanation of antigenic drift. Also, there was an important guiding principle of one-to-many correlation rules for making broad-spectrum vaccines. However, there was no example available to support our findings. Taking the SARS-CoV-2 vaccine as an example, the current multivalent broad-spectrum vaccine still adopts a strategy of combining multiple antigens. Liang et al. developed a broad host SARS-CoV-2 vaccine by incorporating heterologous RBDs from a variety of representative strains as hybrid antigens (34, 35). Zhao et al. designed a new broad host vaccine called pan-vaccine antigen (Span) with cross-clade commonality at assigned sites with high-frequency residues (36). We look forward to developing multivalent broad-spectrum vaccines or rescue mAb in the future based on the one-to-many principle.

However, some observations do not provide an appropriate explanation in this study. For example, the replacement of P6 residues with T, S, and V residues in the HA-tag increased the affinity by 16%-21%. In VSV-tag, the M6 residue was replaced by C and three basic amino acid (R, K, and H) residues further increased the affinity by 12%-23% (**Figures 1A**, **2**).

This study especially quoted a massive quantity of SARS-CoV-2 immune escape data; nevertheless, the main goal of this study was to find the fundamentals of the Ag-Ab recognition process rather than address the problem of SARS-CoV-2 vaccine upgrading to highlight the fundamental principles. Simple linear tag protein mutations and the resulting shift inside the affinity of anti-tag mAbs have been no longer without delay, correlating with the modifications within the SARS-COV-2 Spike protein that is responsible for immunological getaway. To validate this protein-mAb evasion idea in the future, medical examples are required. These verification experiments could not be listed for this study due to budgetary restrictions.

## Conclusion

This study had theoretical importance for future research on antigenic drift and rescue mAb. Furthermore, the following data were summarized:

(I) Ag-Ab interactions were classified into four types: IEM, ADERM, EQM, and IVM, according to antibody binding affinity.
(II) One-to-many interaction rule: For most antibodies and antigenic amino acids, the recognition and binding mode was one-to-many. The observations were verified by the literature data.
(III) IVM and EQM predominance rule: Most of the substitutions with specific antigenic amino acids were IVM and EQM.
(IV) Aryl mutation escape rule: Once aromatic amino acids replaced the antigenic non-aromatic amino acid, the mutation was IEM.

### Limitations

Although supported by experimental and literature data, our study still has some shortcomings. First, the Ag-Ab amino acids interaction pattern did not cover all 20 amino acids due to the absence of certain amino acids in linear epitopes, but the integrated 3D database analysis made up for this shortcoming. Second, the Ag-Ab interaction data derived from the linear protein tag epitopes may not adequately reflect the true spatial information of complete antigen molecules. Third, on account that antigens no longer exactly reflected viruses visible within the actual world, four short protein tags were used. This study did not set out to develop a quick technique or methodology for coping with real-world viral immune escape. However, the fundamental concept of amino acid interaction between Ag and Ab might help us to predict the binding or escape of viruses and Abs in the real world. Based on this approach, our study could draw more attention to combat life-threatening viruses. Therefore, the main task was to verify the validity of this hypothesis in the future by testing contrast antibodies.

## Data availability statement

The original contributions presented in the study are included in the article/**Supplementary Material**. Further inquiries can be directed to the corresponding author.

## Author contributions

MJ: Writing – original draft, Data curation, Methodology, Software, Visualization. CF: Software, Methodology, Validation. YM: Writing – review & editing, Conceptualization, Formal Analysis, Investigation, Project administration, Supervision, Writing – original draft.
