# Supplementary material for: Deciphering the rule of antigen-antibody amino acid interaction
Source: Front Immunol. 2023 Dec 4;14:1269916. doi: 10.3389/fimmu.2023.1269916 (PMC10725943; doi:10.3389/fimmu.2023.1269916)
Supplement: Supplementary file 1 [file DataSheet_1.pdf]

Supplemental Tables

Supplemental Table S1 Sequences of HA-tag and its mutants

Number	sequences
HA-tag	**YPYDVPDYA**
HA01(Y1G)	**GPYDVPDYA**
HA02(P2G)	Y**GYDVPDYA**
HA03(D4G)	YPY**GVPDYA**
HA04(V5G)	YPYD**GPDYA**
HA05(P6G)	YPYDV**GDYA**
HA06(D7G)	YPYDVP**GYA**
HA07(Y8G)	YPYDVPD**GA**
HA08(A9G)	YPYDVPDY**G**
HA09(Y3G)	YP**GDVPDYA**
HA10(Y1E)	**EPYDVPDYA**
HA11(P2E)	Y**EYDVPDYA**
HA12(Y3E)	YP**EDVPDYA**
HA13(D4E)	YPY**EVPDYA**
HA14(V5E)	YPYD**EPDYA**
HA15(P6E)	YPYDV**EDYA**
HA16(D7E)	YPYDVP**EYA**
HA17(Y8E)	YPYDVPD**EA**
HA18(A9E)	YPYDVPDY**E**
HA21(Y1H)	**HPYDVPDYA**
HA22(P2H)	Y**HYDVPDYA**
HA23(Y3H)	YP**HDVPDYA**
HA24(D4H)	YPY**HVPDYA**
HA25(V5H)	YPYD**HPDYA**
HA26(P6H)	YPYDV**HDYA**

HA27(D7H)	YPYDVP**HYA**
HA28(Y8H)	YPYDVPD**HA**
HA29(A9H)	YPYDVPDY**H**
HA31(V5A)	YPYD**APDYA**
HA32(V5T)	YPYD**TPDYA**
HA33(V5K)	YPYD**KPDYA**
HA34(V5R)	YPYD**RPDYA**
HA35(V5Y)	YPYD**YPDYA**
HA36(V5S)	YPYD**SPDYA**
HA37(V5C)	YPYD**CPDYA**
HA38(V5L)	YPYD**LPDYA**
HA39(V5M)	YPYD**MPDYA**
HA40(V5I)	YPYD**IPDYA**
HA41(V5N)	YPYD**NPDYA**
HA42(V5F)	YPYD**FPDYA**
HA43(V5Q)	YPYD**QPDYA**
HA44(V5D)	YPYD**DPDYA**
HA45(V5W)	YPYD**WPDYA**
HA46(V5P)	YPYD**PPDYA**
HA51(P6A)	YPYDV**ADYA**
HA52(P6T)	YPYDV**TDYA**
HA53(P6K)	YPYDV**KDYA**
HA54(P6Y)	YPYDV**YDYA**
HA55(P6S)	YPYDV**SDYA**
HA56(P6C)	YPYDV**CDYA**
HA57(P6V)	YPYDV**VDYA**
HA58(P6L)	YPYDV**LDYA**
HA59(P6M)	YPYDV**MDYA**
HA60(P6I)	YPYDV**IDYA**

HA61(P6W)	YPYDV**WDYA**
HA62(P6F)	YPYDV**FDYA**
HA63(P6Q)	YPYDV**QDYA**
HA64(P6D)	YPYDV**DDYA**
HA65(P6R)	YPYDV**RDYA**
HA66(P6N)	YPYDV**NDYA**
HA82(D4A)	YPY**A**VPDYA
HA83(D4C)	YPY**C**VPDYA
HA84(D4F)	YPY**F**VPDYA
HA85(D4I)	YPY**I**VPDYA
HA87(D4L)	YPY**L**VPDYA
HA88(D4M)	YPY**M**VPDYA
HA90(D4P)	YPY**P**VPDYA
HA93(D4S)	YPY**S**VPDYA
HA95(D4Y)	YPY**Y**VPDYA
HA96(D4W)	YPY**W**VPDYA
HA99(Y3C)	YP**C**DVPDYA
HA100(Y3D)	YP**D**DVPDYA
HA101(Y3F)	YP**F**DVPDYA
HA102(Y3I)	YP**I**DVPDYA
HA103(Y3K)	YP**K**DVPDYA
HA104(Y3L)	YP**L**DVPDYA
HA105(Y3M)	YP**M**DVPDYA
HA106(Y3N)	YP**N**DVPDYA
HA107(Y3P)	YP**P**DVPDYA
HA108(Y3Q)	YP**Q**DVPDYA
HA109(Y3R)	YP**R**DVPDYA
HA110(Y3S)	YP**S**DVPDYA
HA111(Y3T)	YP**T**DVPDYA

HA112(Y3V)	YP**V**DVPDYA
HA113(Y3W)	YP**W**DVPDYA

# The replaced residues were highlighted in red color. To increase the solubility and binding ability of the universal protein tags to the 96-well plate, random peptides of 18 amino acids were fused with the target tag at C-terminus via the GSGSGS linker, respectively.

Supplemental Table S2 Sequences of MYC-tag and its mutants

Number	Sequence
MYC-tag	**EQKLISEEDL**
MYC01(Q2G)	E**G**KLISEEDL
MYC02(L4G)	EQK**G**ISEEDL
MYC03(I5G)	EQKL**G**SEEDL
MYC04(S6G)	EQKLI**G**EEDL
MYC05(E7G)	EQKLIS**G**EDL
MYC06(E8G)	EQKLISE**G**DL
MYC07(L4A)	EQK**A**ISEEDL
MYC08(L4C)	EQK**C**ISEEDL
MYC09(L4D)	EQK**D**ISEEDL
MYC10(L4E)	EQK**E**ISEEDL
MYC12(L4F)	EQK**F**ISEEDL
MYC12(L4H)	EQK**H**ISEEDL
MYC13(L4I)	EQK**I**ISEEDL
MYC14(L4K)	EQK**K**ISEEDL
MYC15(L4M)	EQK**M**ISEEDL
MYC16(L4N)	EQK**N**ISEEDL
MYC17(L4P)	EQK**P**ISEEDL
MYC18(L4Q)	EQK**Q**ISEEDL
MYC19(L4R)	EQK**R**ISEEDL

MYC20(L4S)	EQKSISEEDL
MYC21(L4T)	EQKTISEEDL
MYC22(L4V)	EQKVISEEDL
MYC23(L4W)	EQKWISEEDL
MYC24(L4Y)	EQKYISEEDL
MYC27(I5A)	EQKLASEEDL
MYC28(I5C)	EQKLCSEEDL
MYC29(I5D)	EQKLDSEEDL
MYC30(I5E)	EQKLESEEDL
MYC31(I5F)	EQKLFSEEDL
MYC32(I5H)	EQKLHSEEDL
MYC33(I5K)	EQKLKSEEDL
MYC34(I5L)	EQKLLSEEDL
MYC35(I5M)	EQKLMSEEDL
MYC36(I5N)	EQKLNSEEDL
MYC37(I5P)	EQKLPSEEDL
MYC38(I5Q)	EQKLQSEEDL
MYC39(I5R)	EQKLRSEEDL
MYC40(I5S)	EQKLSSEEDL
MYC41(I5T)	EQKLTSEEDL
MYC42(I5V)	EQKLVSEEDL

# The replaced residues were highlighted in red color. To increase the solubility and binding ability of the universal protein tags to the 96-well plate, random peptides of 18 amino acids were fused with the target tag at C-terminus via the GSGSGS linker, respectively.

Supplemental Table S3 Sequences of Flag-tag and its mutants

Number	Sequence
**Flag**-tag	**DYKDDDDK**

Flag01(K3G)	DY**G**DDDDK
Flag02(K8G)	DYKDDDD**G**
Flag03(K3A)	DY**A**DDDDK
Flag04(K3C)	DY**C**DDDDK
Flag05(K3D)	DY**D**DDDDK
Flag06(K3E)	DY**E**DDDDK
Flag07(K3F)	DY**F**DDDDK
Flag08(K3H)	DY**H**DDDDK
Flag09(K3I)	DY**I**DDDDK
Flag10(K3L)	DY**L**DDDDK
Flag11(K3M)	DY**M**DDDDK
Flag12(K3N)	DY**N**DDDDK
Flag13(K3P)	DY**P**DDDDK
Flag14(K3Q)	DY**Q**DDDDK
Flag15(K3R)	DY**R**DDDDK
Flag16(K3S)	DY**S**DDDDK
Flag17(K3T)	DY**T**DDDDK
Flag18(K3V)	DY**V**DDDDK
Flag19(K3W)	DY**W**DDDDK
Flag20(K3Y)	DY**Y**DDDDK

# The replaced residues were highlighted in red color. To increase the solubility and binding ability of the universal protein tags to the 96-well plate, random peptides of 18 amino acids were fused with the target tag at C-terminus via the GSGSGS linker, respectively.

Supplemental Table S4 Sequences of VSV-tag and its mutants

Number	Sequence
VSV-tag	**YTDIEMNRLGK**
VSV01(T2G)	Y**G**DIEMNRLGK

VSV02(I4G)	YTD**G**EMNRLGK
VSV03(M6G)	YTDIE**G**NRLGK
VSV04(N7G)	YTDIEM**G**RLGK
VSV05(R8G)	YTDIEMN**G**LGK
VSV06(T2A)	Y**A**DIEMNRLGK
VSV07(T2C)	Y**C**DIEMNRLGK
VSV08(T2D)	Y**D**DIEMNRLGK
VSV09(T2E)	Y**E**DIEMNRLGK
VSV10(T2F)	Y**F**DIEMNRLGK
VSV11(T2H)	Y**H**DIEMNRLGK
VSV12(T2I)	Y**I**DIEMNRLGK
VSV13(T2K)	Y**K**DIEMNRLGK
VSV14(T2L)	Y**L**DIEMNRLGK
VSV15(T2M)	Y**M**DIEMNRLGK
VSV16(T2N)	Y**N**DIEMNRLGK
VSV17(T2P)	Y**P**DIEMNRLGK
VSV18(T2Q)	Y**Q**DIEMNRLGK
VSV19(T2R)	Y**R**DIEMNRLGK
VSV20(T2S)	Y**S**DIEMNRLGK
VSV21(T2V)	Y**V**DIEMNRLGK
VSV22(T2W)	Y**W**DIEMNRLGK
VSV23(T2Y)	Y**Y**DIEMNRLGK
VSV26(M6A)	YTDIE**A**NRLGK
VSV27(M6C)	YTDIE**C**NRLGK
VSV28(M6D)	YTDIE**D**NRLGK
VSV29(M6E)	YTDIE**E**NRLGK
VSV30(M6F)	YTDIE**F**NRLGK
VSV31(M6H)	YTDIE**H**NRLGK
VSV32(M6I)	YTDIE**I**NRLGK

VSV33(M6K)	YTDIE**K**NRLGK
VSV34(M6L)	YTDIE**L**NRLGK
VSV35(M6N)	YTDIE**N**NRLGK
VSV36(M6P)	YTDIE**P**NRLGK
VSV37(M6Q)	YTDIE**Q**NRLGK
VSV38(M6R)	YTDIE**R**NRLGK
VSV39(M6S)	YTDIE**S**NRLGK
VSV40(M6T)	YTDIE**T**NRLGK
VSV41(M6V)	YTDIE**V**NRLGK
VSV42(M6W)	YTDIE**W**NRLGK
VSV43(M6Y)	YTDIE**Y**NRLGK

# The replaced residues were highlighted in red color. To increase the solubility and binding ability of the universal protein tags to the 96-well plate, random peptides of 18 amino acids were fused with the target tag at C-terminus via the GSGSGS linker, respectively.

Supplemental Figures.

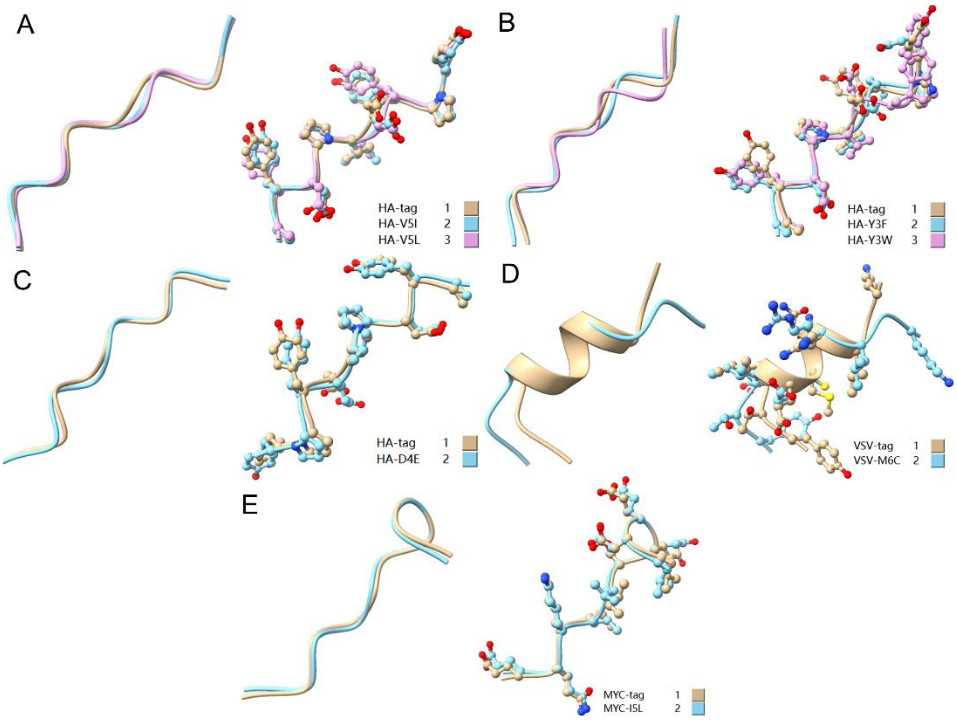

Figure S1. Comparison of amino acid mutant with similar physicochemical property residues.

(A) Comparison of HA-tag, HA-V5I, and HA-V5L; (B) Comparison of HA-tag, HAY3W, and HA-Y3F; (C) Comparison of HA-tag and HA-D4E; (D) Comparison of VSV-tag and VSV-M6C; (E) MYC-tag and MYC-I5L. The models were predicted using the software AlphaFold.

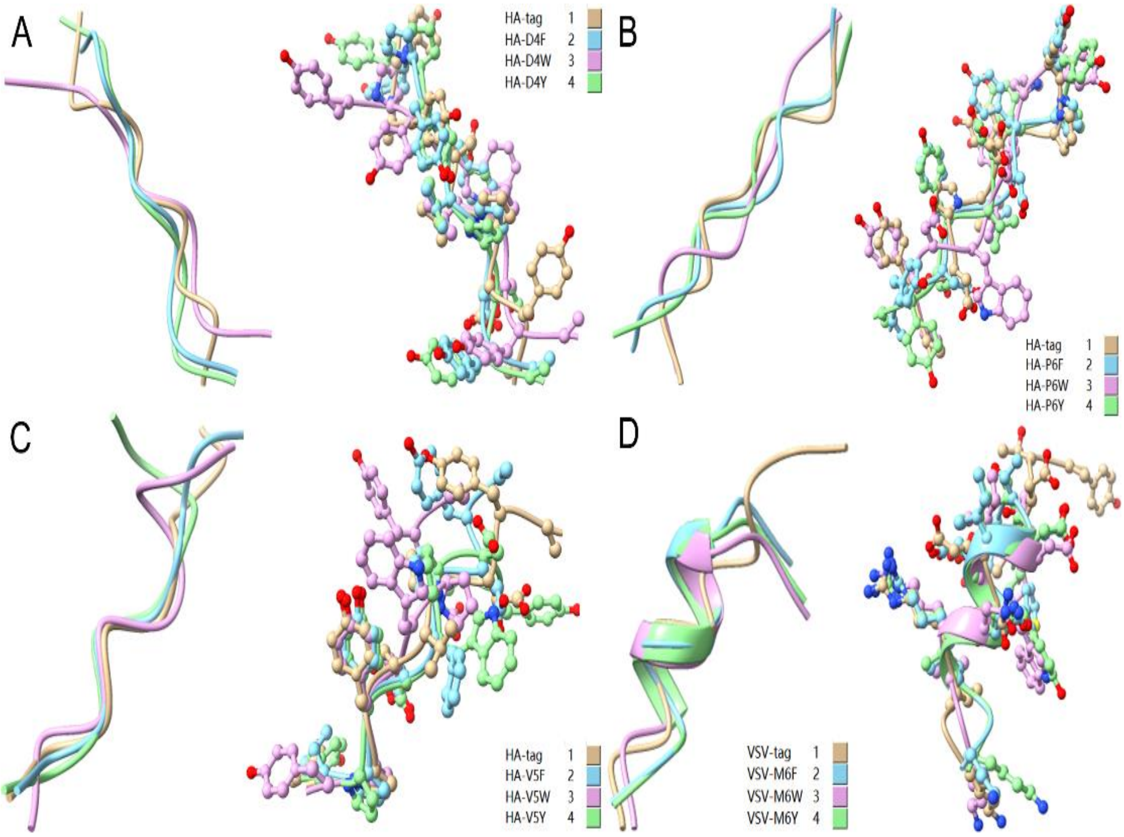

Figure S2. Comparison of aromatic amino acid substitution mutants.

(A) Comparison of HA-tag, HA-D4F, HA-D4Y, and HA-D4W; (B) Comparison of HA-tag, HA-V5F, HA-V5Y, and HA-V5W; (C) Comparison of HA-tag, HA-P6F, HA-P6Y, and HA-P6W; (D) Comparison of VSV-tag, VSV-M6F, VSV-M6Y, and VSV-M6W. The models were predicted using the software AlphaFold.
